# Implications of the microbiome and metabolic intermediaries produced by bacteria in breast cancer

**DOI:** 10.1590/1678-4685-GMB-2023-0316

**Published:** 2024-07-22

**Authors:** Vívian D’Afonseca, Elizabeth Valdés Muñoz, Alan López Leal, Patricio Maximiliano Adrián Suazo Soto, Cristóbal Parra-Cid

**Affiliations:** 1 Universidad Católica del Maule, Facultad de Medicina, Departamento de Ciencias Preclinicas, Laboratorio de Microbiología y Parasitología, Talca, Chile.; 2 Universidad Católica del Maule, Centro de Biotecnología de los Recursos Naturales (CENBIO), Programa de Doctorado en Biotecnología Traslacional, Talca, Chile.; 3 Universidad Católica del Maule, Centro de Biotecnología de los Recursos Naturales (CENBIO), Talca, Chile.; 4 Millennium Initiative for Collaborative Research on Bacterial Resistance (MICROB-R), Santiago, Chile.; 5 Universitat de Barcelona, Facultad de Farmacia y Ciencias de la Alimentación, Programa de Máster en Biotecnología Molecular, Barcelona, España.

**Keywords:** Microbiome, cancer, breast cancer, dysbiosis, bacterial metabolites

## Abstract

The breast microbiome presents a diverse microbial community that could affects health and disease states, in the context of breast cancer. Sequencing technologies have allowed describing the diversity and abundance of microbial communities among individuals. The complex tumoral microenvironment that includes the microbial composition could influence tumor growth. The imbalance of diversity and abundance inside the microbial community, known as dysbiosis plays a crucial role in this context. One the most prevalent bacterial genera described in breast invasive carcinoma are *Bacillus, Pseudomonas, Brevibacillus, Mycobacterium, Thermoviga, Acinetobacter, Corynebacterium, Paenibacillus, Ensifer,* and *Bacteroides*. *Paenibacills* genus shows a relation with patient survival. When the *Paenibacills* genus increases its abundance in patients with breast cancer, the survival probability decreases. Within this dysbiotic environment, various bacterial metabolites could play a pivotal role in the progression and modulation of breast cancer. Key bacterial metabolites, such as cadaverine, lipopolysaccharides (LPS), and trimethylamine N-oxide (TMAO), have been found to exhibit potential interactions within breast tissue microenvironments. Understanding the intricate relationships between dysbiosis and these metabolites in breast cancer may open new avenues for diagnostic biomarkers and therapeutic targets. Further research is essential to unravel the specific roles and mechanisms of these microbial metabolites in breast cancer progression.

## Breast cancer

According to the World Health Organization (WHO), breast cancer is considered one of the most prevalent tumors worldwide, with over 2.2 million new cases reported and more than 685,000 women deaths in 2020 (WHO, 2023). Breast cancer is a non-communicable chronic ailment that originates when cells within breast tissue lose their ability to regulate their normal growth and division, resulting in uncontrolled proliferation. This unregulated cell multiplication leads to aberrant proliferation, marking the initiation of a carcinogenesis process. Breast cancer is an intricately heterogeneous disease, comprising established subtypes with significant variability in the progression of the disease within each subtype. Presently, breast carcinoma is categorized into four molecular classes: luminal A, luminal B, *HER2* (Human epidermal growth factor receptor 2), and triple-negative (TN) with basal and non-basal phenotypes (Calderón Del Valle and Gallón Villega, 2012; Fernández and Reigosa, 2016).

The majority of breast cancer cases are sporadic, meaning they lack a specific hereditary pattern, with genetic, epigenetic, and genomic changes predominantly occurring in somatic cells. It is estimated that only 5 to 10% of breast carcinomas are considered hereditary syndromes, with these alterations potentially being passed between generations as an autosomal dominant disease (Calderón Del Valle and Gallón Villega, 2012). Syndromes associated with this type of tumor are characterized by early onset, vertical transmission of genetic risk factors, bilateral tumor presentation in both breasts and instances of other cancers within the same family (Calderón Del Valle and Gallón Villega, 2012; Fernández and Reigosa, 2016). The hereditary pattern of breast carcinoma is linked to various high-penetrance genes, such as *BRCA1* and *BRCA2*.

However, the genesis of many cancerous processes cannot be solely attributed to genetic changes, as environmental factors play a substantial role in these mechanisms. The microbiome is one such factor (Álvarez-Mercado et al., 2023). The relationship of the microbiome with the development of specific cancers such as colorectal and gastric cancer has been broadly evidenced, however, there has been a growing focus on the proposed link between the microbiome and breast cancer. This review explores the recent association between the microbiome and breast cancer, acknowledging the emerging dimensions of cancer hallmarks, particularly in the context of polymorphic microbiomes (Hanahan, 2023).

## Microbiome

The human microbiome comprises a complex assembly of microorganisms - bacteria, viruses, fungi, protozoa, and archaea - that coexist in various regions of the human body, including the skin, oral mucosa, vagina, lungs, and predominantly the gastrointestinal system (Karen, 2008). These microorganisms exhibit a spectrum of effects, ranging from beneficial to harmful or neutral (Cheng et al., 2020), collectively contributing to the body’s overall equilibrium, including reinforcing the body’s defenses and facilitating nutrient metabolism. The gastrointestinal tract hosts the most expansive and diverse human-associated microbiome, housing trillions of microorganism cells and an extensive array of species (Sender et al., 2018). Alterations in the diverse landscape of the gut microbiome, known as “dysbiosis”, are recognized as pivotal factors in the development of both metabolic diseases and cancer. Recent studies have highlighted a connection between the intestinal microbiome and various intestinal diseases, notably colorectal cancer (CRC). While these changes in the gut microbiome, observed in individuals with CRC, are not definitively causal in carcinogenesis, they are substantive enough to serve as diagnostic indicators and, in certain cases, prognostic markers for this cancer (Saus et al., 2019).

For instance, specific bacterial species, such as *Fusobacterium nucleatum*, *Bacteroides fragilis*, and *Enterococcus faecalis*, have been associated with consequential changes in the intestinal epithelium, instigating an inflammatory response that can incite DNA damage and local cell proliferation (Saus et al., 2019). Moreover, CRC patients exhibit a heightened presence of proinflammatory opportunistic bacteria and microbes associated with metabolic disorders. Species like *F. nucleatum, Streptococcus gallolyticus*, *Escherichia coli*, *B. fragilis*, and *E. faecalis* are predominant in collected fecal samples from CRC patients. At the same time, genera like *Roseburia, Clostridium, Faecalibacterium*, and *Bifidobacterium* are comparatively scarce in individuals with CRC (Saus *et al.*, 2019). These microbial shifts are observed as significant contributors to the pathogenesis of CRC.

## Dysbiosis in the context of cancer development

Dysbiosis is a state characterized by persistent imbalance in the microbiome, primarily in the gut, which typically plays a beneficial role in maintaining the body’s health (DeGruttola et al., 2016). This imbalance can give rise to various health conditions including obesity, inflammatory bowel disease (IBD), and even cancer (DeGruttola *et al.*, 2016). Dysbiosis involves a notable alteration in the composition of the microbiome, surpassing what is considered normal for a specific group of subjects under study and it is typically characterized by three key elements. First, an increase in harmful bacteria (Zhang et al., 2022). Second, a decrease in beneficial bacteria (Korem et al., 2015), and third a reduction in microbiome diversity (Kostic et al., 2015). Additionally, it can be triggered by a myriad of factors (Levy et al., 2017), including infections and inflammations (Zhang *et al.*, 2022), dietary choices, and exposure to foreign chemicals (Norman et al., 2015; Sonnenburg et al., 2016), genetic influences (Levy *et al.*, 2015), and hereditary predispositions (Stappenbeck and Virgin, 2016). 

The complex interplay of the microbiome significantly affects host cell growth, programmed cell death, immune response modulation, and the metabolism of indigestible dietary components, xenobiotics, and pharmaceuticals (Parida and Sharma, 2019). Several studies have attempted to define the composition of a core healthy microbiome to understand the pathological mechanisms underlying diseases such as cancer and inflammatory disorders within dysbiotic scenarios. While only a few specific microbes are established as direct causative agents of cancer (e.g., *Helicobacter pylori*), numerous microbes appear to contribute to cancer progression through modulation of the host’s immune system. Certain microbes possess immunostimulatory properties that hold significant implications for cancer development and the immune surveillance of tumors (Sepich-Poore et al., 2021). An exemplary case is a strong association between the Gram-negative bacteria *F. nucleatum* and colorectal cancer, evident in abundance within tumor tissues and pre-cancerous adenomas, particularly in high-grade dysplasia tumors (Sheflin et al., 2014). The role of the microbiome extends beyond solid tumors to encompass cancers such as leukemia. Preclinical investigations in mice have revealed a probable correlation between specific genetic predispositions leading to leukemia and consequential alterations in the intestinal microbiome in these animals (Dueñas *et al.*, 2020). Nycz et al. (2018) scrutinized stool samples from 42 pediatric leukemia patients at various treatment stages unveiling microbial changes over time and under diverse treatment conditions (Nycz *et al.*, 2018). Similar studies enabled the observation of gut bacterial composition alterations as treatment progressed in pediatric leukemia patients (Wang et al., 2014; Tidjani *et al.*, 2016). For instance, bacterial groups like *Clostridiaceae* and *Bacteroidaceae* dominate in healthy children (Wang *et al.*, 2014; Tidjani *et al.*, 2016), but in cases of acute lymphoblastic leukemia, the *Bacteroidaceae* groups are more abundant at diagnosis while the *Clostridiaceae* and *Lachnospiraceae* groups decrease (Tidjani *et al.*, 2016). While studies have not yet demonstrated that changes in individual’s microbiota composition leads to the development of leukemia, for example, it is already known that the microbiota could be altered with the progression of treatments as a side effect or rather could be affected by genetics predispositions.

These findings underscore the substantial influence of dysbiosis in shaping the microbiome’s association with cancer development, whether it involves solid tumors such as colorectal cancer or hematologic malignancies like leukemia. Understanding these dynamic interactions between the microbiome and cancer progression is vital for advancing potential therapeutic strategies and diagnostic approaches.

## Breast microbiome and breast cancer

In the context of breast cancer, the diverse and distinctive bacterial community present in the female mammary gland stands out in comparison to other bodily sites. Notably, this community remains independent of age, pregnancy, or geographical origin (Urbaniak et al., 2014). Emerging evidence strongly suggests that part of the breast tissue microbiome originates from translocation either from the gastrointestinal tract or through the skin, primarily via the areola-nipple openings, oral-nipple contact during breastfeeding, or potentially even through sexual contact. It is theorized that this mammary microbiome contributes significantly to the preservation of healthy breast tissue by, for instance, activating resident immune cells. Additionally, the specific type of bacteria and their metabolic activity, particularly their ability to degrade potential carcinogens, might play a crucial role in this context (Urbaniak *et al.*, 2014).

Advanced sequencing technologies and insights gained from the Human Microbiome Project have revealed that the diversity and abundance of microbial communities vary significantly among individuals (Human Microbiome Project Consortium, 2012). There is a prevailing hypothesis that the breast microbiome could directly influence the risk of developing breast cancer. While this hypothesis suggests various pathways for disease alterations and progression, it does not conclusively identify a specific microbial pattern responsible for breast carcinogenesis (Urbaniak et al., 2014; Urbaniak *et al.*, 2016). 

The breast shows a sophisticated microenvironment that comprises complex systems including epithelial, interstitial, and mucosal immune systems (Going and Moffat, 2004). Microbial exposure induces the modulation of the immune system and its mucosa, where inflammation processes could happen facing changes in the microenvironment present in those tissues induced by bacterial infections (Schwabe and Jobin, 2013). Thus, the presence of altered immune responses in the breast microenvironment could be through the influence of the mammary microbial community and its deviations.

**Figure 1 -:**
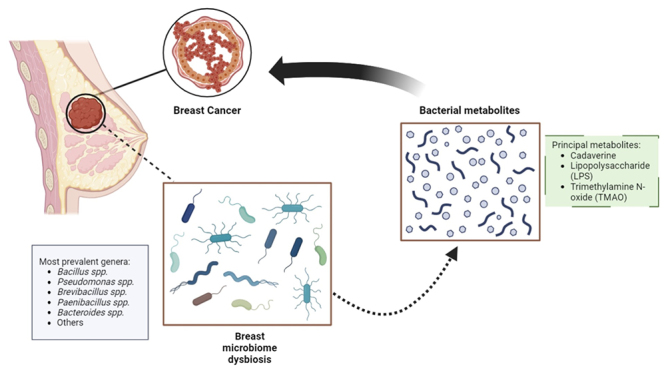
Bacterial composition commonly found in breast cancer tissue and bacterial metabolites produced in breast dysbiosis conditions.

Currently, normal breast tissue hosts a dominant microbial community inclusive of *Proteobacteria*, *Firmicutes* (Urbaniak et al., 2014), *Sphingomonas yanoikuyae* (Xuan et al., 2014)*, Actinobacteria* (Thompson et al., 2017)*, Methylobacterium* (Wang et al., 2017)*, Ralstonia* (Constantini et al., 2018)*, Bacteroidaceae* (Meng et al., 2018)*, Prevotella, Lactococcus, Streptococcus, Corynebacterium, Staphylococcus* (Urbaniak *et al.*, 2016), and an unclassified genus of the family *Sphingomonadaceae* (Chan et al., 2016). The higher prevalence of *Proteobacteria* and *Firmicutes* in comparison to other taxonomic groups could stem from microbial adaptation to the fatty acid-rich tissue environment (Figure 1).

## Dysbiosis of the microbiome in breast cancer

The microenvironment in and around tumors encompasses a diverse array of cell types, including the microbiome. The physiological and pathological changes occurring in these cells, as well as the microbial composition, significantly influence tumor growth. Dysbiosis, characterized by the disruption of normal microbial community function and the breakdown of symbiotic relationships within this community, plays a pivotal role in this context.

An analysis conducted by Xuan et al. (2014) highlighted important findings regarding bacterial quantity between normal tissue and breast cancer patients. Interestingly, they determined that the number of Operational Taxonomic Units (OTUs) remained consistent between normal tissue and tumor, indicating no significant variations. However, it is notable that breast tumor tissue exhibited significantly reduced quantities of bacteria, and the community uniformity differed significantly (p = 0.01). From the 1614 OTUs detected, 11 were differentially abundant (p < 0.05), with eight more prevalent in paired normal tissue and three more abundant in tumor tissue (Xuan *et al.*, 2014). The study observed notable differences in the genera *Methylbacterium* and *Sphingomonas* between adjacent tissue and tumor tissue, indicating a potential role for these bacteria in cancer development. *Methylobacterium radiotolerans* was found to be the most prevalent bacteria in tumor tissue, present in 100% of the samples. Conversely, *Sphingomonas yanoikuyae* was found in 95% of the samples and exhibited significantly higher absolute levels in normal tissue. Intriguingly, *Sphingomonas yanoikuyae* was absent in the corresponding tumor tissue. The relative abundances of these two bacterial species inversely correlated in normal breast tissue but not in tumor tissue, suggesting a link between dysbiosis and breast cancer. Notably, *M. radiotolerans* was present in all samples, with its absolute levels showing no significant variance between normal tissue and tumor tissue. This suggests that the higher relative abundance of *M. radiotolerans* in the tumor reflects a decrease in other co-existing bacteria rather than an increase in the organism’s absolute levels (Xuan *et al.*, 2014).

## Understanding the breast microbiome in breast cancer studies

Numerous studies have analyzed the breast microbiome highlighting the predominance of the *Proteobacteria* and *Firmicutes* phyla, underscoring their substantial presence, although with some variations. Urbaniak et al. (2014) conducted an extensive investigation to discern the specific microbiome within breast tissue. Examining a sizeable cohort of women of Irish and Canadian descent with and without breast cancer, they uncovered a diverse bacterial population across all tissues studied. Among the most abundant phyla observed in breast tissue were *Proteobacteria* and *Firmicutes*, which these two groups of bacteria were more representative than other taxonomic groups. The authors postulated that these findings could be attributed to a probable microbial adaptation to the fatty acid-rich environment of breast tissue. Notably, the principal OTUs were associated with seven distinct phyla: *Proteobacteria*, *Firmicutes*, *Actinobacteria*, *Bacteroidetes*, *Deinococcus thermus*, *Verrucomicrobia*, and *Fusobacteria*, with *Proteobacteria* being the most prevalent, followed by *Firmicutes*.

In a subsequent study by Urbaniak et al. (2016), they found differing bacterial profiles in breast tissue among healthy women and those diagnosed with breast cancer. Similarly, Hieken et al. (2016) noted significant distinctions in the breast microbiome of women with benign conditions compared to those with malignant tumors. Comparing adjacent tissue from women with breast cancer to that of healthy counterparts, they identified significantly higher relative abundances of specific bacterial genera in each group. Healthy patients exhibited a prevalence of *Prevotella*, *Lactococcus*, *Streptococcus*, *Corynebacterium*, and *Micrococcus*, while breast cancer patients have showcased higher levels of *Bacillus*, *Staphylococcus*, *Enterobacteriaceae*, *Comamondaceae*, and *Bacteroidetes*. Notably, the latter group’s bacteria demonstrated the ability to induce DNA damage *in vitro* (Hieken *et al.*, 2016).

Thompson et al. (2017) characterized the breast microbiome in 668 breast tumor tissues and 72 adjacent non-cancerous tissues, unveiling potential alterations in the microbial composition among different disease subtypes. Predominant phyla in tumor sites included *Proteobacteria* (48.0%), *Actinobacteria* (26.3%), and *Firmicutes* (16.2%), aligning with prior findings. Differentially abundant species observed in tumor samples were *Mycobacterium fortuitum* and *Mycobacterium phlei*. Moreover, *Proteobacteria* exhibited a higher prevalence in tumor tissues, whereas *Actinobacteria* were more prevalent in non-cancerous adjacent tissue samples (Thompson *et al.*, 2017).

Another study conducted by Kim et al. (2021) showed the potential involvement of the microbiome in breast tumor progression. Analyzing 114 samples from Korean breast cancer patients - comprising tumor, adjacent normal, and lymph node tissues - they noted microbial divisions into two clusters without discernible differences among the tissues studied. Notably, the microbiome’s categorization into these clusters was correlated with clinicopathologic factors like the risk of regional recurrence, showing the potential impact of *Enterococcus* spp. in shaping these differences (Kim *et al.*, 2021).

Tzeng et al. (2021) employed 16S rRNA gene sequencing to analyze the human breast tissue microbiome across 221 breast cancer patients, 18 individuals prone to breast cancer, and 69 control subjects, revealing substantial insights. Their findings highlighted noteworthy differences in the relative abundance of multiple bacterial genera when stratified across distinct breast tissue types, cancer stages, grades, histological subtypes, and other clinical factors. Of particular significance was the absence of *Anaerococcus*, *Caulobacter*, and *Streptococcus* - found prevalent in benign tissue - in the cancer-associated tissue. Furthermore, the investigation identified *Proteobacteria* as the dominant bacterial phylum in breast tissues, followed by *Firmicutes* and *Actinobacteria*. Their analysis unveiled a lower abundance of *Enterobacteriaceae* alongside a higher prevalence of *Corynebacterium*, *Lactococcus*, and *Streptococcus* in breast tissue obtained from healthy individuals instead of those afflicted by cancer. These findings contribute significantly to our understanding of the distinct microbial compositions associated with breast cancer, offering potential avenues for further research and clinical implications.

The Bacteria in Cancer (BIC) Database harbors data from The Cancer Genome Atlas (TCGA), which includes bacteria expression profiles from whole genome sequencing (WGS), and whole exon sequencing (WXS) (Kai-Pu et al., 2023). This database shows the ten most prevalent bacterial genera in breast invasive carcinoma (BRCA) such as *Bacillus, Pseudomonas, Brevibacillus, Mycobacterium, Thermoviga, Acinetobacter, Corynebacterium, Paenibacillus, Ensifer* and *Bacteroides*. Regarding clinical importance, two genera stand out from those mentioned above *Corynebacterium* and *Paenibacillus*. *Corynebacterium* genus shows that relative abundance is very expressive in normal tissue in comparison to the tumor tissue (p = 7.105e-18). *Paenibacills* genus shows a relation with patient survival. When the *Paenibacills* genus increases its abundance in patients with breast cancer, the survival probability survival decreases to around 30% (p = 1.545e-02).

## Bacterial metabolites and their potential role in cancer

The evolution of scientific inquiry has tirelessly sought to discover, quantify, and define analytes - commonly recognized as cancer biomarkers - pivotal in the clinical landscape. Notably, these biomarkers, like CA15-3/CA27.29, CA27.29, *BRCA1*, and *BRCA2*, are detectable in various bodily fluids and hold substantial clinical utility, particularly in the context of breast cancer diagnosis and prognosis (National Cancer Institute, 2023).

**Figure 2 -:**
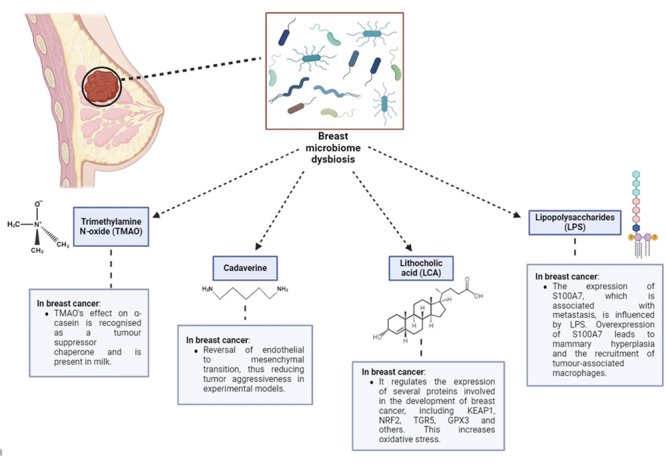
Description of bacterial metabolites produced in breast microbiome dysbiosis.

Breast cancer development is a multistep process that includes multiple oncopathological and inflammatory processes. These intricate mechanisms, when disrupted by dysbiosis, could induce fluctuations in the production of certain metabolites. These metabolites might crucially affect the modulation of breast cancer. Conversely, maintaining a state of intestinal microbiome homeostasis appears to trigger the release of metabolites exhibiting anti-metastatic potential - a promising pathway for potential therapeutic ways (Figure 2). Consequently, the exploration of microbiome-generated metabolites has emerged as an area of scientific interest. Their potential interactions with transcriptional, epigenetic, and metabolic processes within oncology present a captivating frontier for further investigation (Luu and Visekruna, 2021).

A primary signaling conduit linking the microbiome and the host involves the secretion of microbial metabolites that traverse the circulatory system and are directed to specific target cells. (Burcelin et al., 2013). Functionally similar to human hormones, these microbial metabolites exhibit a capacity for biological transmission and action. Moreover, these compounds infiltrate the circulation, influencing and modulating the intestine and other local environments, thereby influencing their function and dynamics (Mikó et al., 2019). This perspective highlights the important role of microbial metabolites in cancer modulation. Its potential effects on diverse biological processes across oncological domains underscore the need for comprehensive exploration and understanding within this complex interplay.

## Trimethylamine N-oxide (TMAO)

Various microbial species, particularly *Desulfovibrio* and *Desulfovibrio* desulfuricans, are known for their ability to convert dietary components like choline into Trimethylamine (TMA) through specific enzymatic pathways, ultimately leading to the production of Trimethylamine N-oxide (TMAO) (Zeisel and Warrier, 2017). This conversion typically occurs following the intake of foods rich in choline and L-carnitine, such as red meat or eggs, which serve as primary sources for these precursors (Demarquoy et al., 2004).

TMAO has been attributed with diverse biological functions, including countering the denaturing effects of pH, elevating osmotic pressure, and stabilizing proteins similar to a molecular chaperone. Additionally, it has implications for lipid metabolism, modulating oxidative stress (Zeisel and Warrier, 2017), and potentially affecting the anti-tumoral immune response mediated by CD8+ T cells (Wang et al., 2022). Specifically in breast cancer, TMAO’s influence on α-casein is recognized as a tumor-suppressing chaperone present in the milk of various mammals (Bhat et al., 2017). This interaction underscores the multifaceted impact of TMAO on cellular mechanisms relevant to breast cancer development and progression, suggesting a potential pathway for further exploration in understanding its specific role in oncological processes.

## Cadaverine

Cadaverine, also recognized as 1,5-diaminopentane, is a natural polyamine generated by the decarboxylation of L-lysine facilitated by lysine decarboxylase, a specific enzyme. This molecule is naturally present in a wide spectrum of both prokaryotic and eukaryotic organisms. The compound exhibits diverse biological properties and holds significant importance in cell survival, particularly in acidic environments, offering protection to cells in anaerobic conditions lacking inorganic phosphate (Pi) (Moreau, 2007). While human cells can also produce cadaverine, bacterial synthesis predominantly contributes to its presence. Notably, various intestinal bacteria such as *Shigella flexneri, Shigella sonnei, Escherichia coli*, and the *Streptococcus* genus are known to express enzymes involved in its biosynthesis (de las Rivas et al., 2006).

Remarkably, studies by Kovács et al. (2019a) observed a reduction in cadaverine levels within the intestinal environment associated with breast cancer development. Intriguingly, in experimental models involving rats transplanted with 4T1 breast cancer cell lines, administration of cadaverine (at 500 nmol/kg) contributed to the reversal of endothelial to mesenchymal transition, thus reducing tumor aggressiveness (Kovács *et al.*, 2019a). This insight implies that dysbiosis in the gut microbiome may potentially diminish agents such as cadaverine, which could otherwise play a protective role against processes associated with carcinogenesis. However, additional research in this area is necessary to uncover direct relationships between cadaverine and its impact on cancer pathways.

## Lithocholic Acid (LCA)

Lithocholic acid (LCA) is a secondary bile acid produced through the enzymatic activity of 7α/β-hydroxysteroid dehydroxylase (*baiH* gene), playing a cytostatic role in breast cancer. Synthesized by the dehydroxylation of chenodeoxycholic acid (CDCA) and ursodeoxycholic acid (UDCA) at position 7 (Long et al., 2017), LCA is primarily generated by anaerobic bacteria, particularly *Clostridiales*, which facilitate the transformation of bile acids. The genes responsible for the degradation of secondary bile acids are part of the bile acid-inducible (*bai*) operon (Ridlon et al., 2006).

LCA exerts anticancer effects through the Takeda G protein-coupled receptor 5 (TGR5). Research conducted by Mikó et al. (2018) revealed that patients diagnosed with early-stage breast cancer exhibited reduced serum levels of lithocholic acid compared to the control group. This reduction in LCA levels, along with variations in bile acid ratios and decreased expression of the *baiH* gene in fecal DNA, suggests the diminished generation of LCA by the intestinal microbiome in early-stage breast cancer (Mikó *et al.*, 2018).

Furthermore, Kovács et al. (2019b) demonstrated that the application of LCA to breast cancer cells resulted in increased expression of Kelch-like ECH-associated protein 1 (KEAP1) and reduced expression of nuclear factor 2 (NRF2). This was achieved via the activation of TGR5 and constitutive androstane receptor (CAR), affecting antioxidant enzyme expression, such as glutathione peroxidase 3 (GPX3), and leading to increased oxidative stress. Pharmacological induction of NRF2 with antioxidants reversed these effects, suggesting the cytostatic impact of LCA due to the imbalance between pro- and antioxidants. As breast cancer progressed, components of the cytostatic pathway triggered by LCA displayed gradual reduction, and this loss was associated with a poor prognosis (Kovács *et al.*, 2019b).

## Lipopolysaccharides (LPS)

Studies that analyze the implications of intratumoral bacteria in tumorigenesis, particularly through DNA damage and tumor progression, have increased in the last decade. Specific bacteria, notably those in the *Enterobacteriaceae* family producing colibactin, have been associated with causing DNA damage and promoting tumorigenesis (Nougayrède et al., 2006; Pleguezuelos-Manzano et al., 2020). While mammary tissues host various commensal bacteria, the link between mammary tumor growth and differential bacterial distribution remains largely unexplored. In a metagenomic analysis conducted by Wilkie et al. (2022) employing a mouse model to assess the microbiome’s association with breast tumor growth, several key findings emerged (Wilkie *et al.*, 2022). The study revealed a substantial increase in Gram-negative bacterial populations in late-stage tumors (LST) and late-stage tumors with dextran sodium sulfate (LSTDSS) compared to control skin samples or early-stage mammary tumors. Notably, higher LPS amounts were detected in the control samples. Furthermore, an increased abundance of Gram-negative bacterial populations was observed in LST and LSTDSS mammary tumors, with no significant difference in abundance between them. Importantly, the study showcased the influence of LPS on the expression of S100A7 (S100 calcium-binding protein A7 or psoriasin), a microbicide protein associated with breast cancer progression and metastasis. Overexpression of S100A7 induced mammary gland hyperplasia and recruited tumor-associated macrophages, and this study highlighted a novel role of LPS in driving S100A7 expression. The findings imply the modulation of the expression of TLR4 and RAGE in invasive breast cancers (Wilkie *et al.*, 2022).

## Risk of describing microbiome studies

It is important to emphasize the risks that may arise from trials using next-generation sequencing techniques in microbiome studies, which must be approached with the utmost caution, always aiming to use blank samples and minimize any contamination caused by sample manipulation that could affect the microbiome composition.

## Conclusion

Understanding the intricate relationship between the microbiome, dysbiosis, and associated bacterial metabolites like LPS, cadaverine, and TMAO could be pivotal in comprehending breast cancer progression. The complex interaction between the microbiome and the host influences various physiological processes, immune responses and metabolic pathways, mainly when there is an imbalance in the microorganisms of this community that leads to dysbiosis. Furthermore, dysbiosis has been correlated with pathological processes, including breast cancer, underscoring the importance of investigating microbial alterations.

Additionally, metabolites produced by the microbiome, such as LPS, cadaverine, and TMAO, have shown the potential to influence molecular, metabolic, and immunological processes, thereby potentially impacting breast cancer pathogenesis. LPS has been associated with S100A7 expression and tumor progression, while metabolites like cadaverine and TMAO exhibit complex interactions with cancer cells and tumor microenvironments, influencing cellular behavior and tumor growth.

The study of these components provides valuable information on potential diagnostic biomarkers, therapeutic targets, and understanding of the intrinsic mechanisms of breast cancer. A deeper exploration of these microbiome-related factors and metabolites holds promise for unveiling novel pathways in breast cancer research, potentially leading to innovative diagnostic methods and therapeutic interventions.
